# Spatial Pattern Evolution and Influencing Factors on Agricultural Non-Point Source Pollution in Small Town Areas under the Background of Rapid Industrialization

**DOI:** 10.3390/ijerph20032667

**Published:** 2023-02-02

**Authors:** Mingtao Yan, Jianji Zhao, Jiajun Qiao, Dong Han, Qiankun Zhu, Yang Yang, Qi Liu, Zhipeng Wang

**Affiliations:** 1 Key Research Institute of Yellow River Civilization and Sustainable Development, Henan University, Kaifeng 475001, China; 2 Collaborative Innovation Center on Yellow River Civilization Jointly Built by Henan Province and Ministry of Education, Henan University, Kaifeng 475001, China; 3 College of Geography and Environmental Science, Henan University, Kaifeng 475001, China; 4 Key Laboratory of Geospatial Technology for the Middle and Lower Yellow River Regions, Henan University, Ministry of Education, Kaifeng 475004, China

**Keywords:** rapid industrialization, small towns, agricultural non-point source pollution, spatial spillover effects, spatial stratified heterogeneity

## Abstract

To promote sustainable agricultural development in small town areas during rapid industrialization, it is important to study the evolution of agricultural non-point source pollution (ANSP) and its influencing factors in small town areas in the context of rapid industrialization. The non-point source inventory method was used to study the characteristics of ANSP evolution in 14 small town areas in Gongyi City from 2002 to 2019. Using the spatial Durbin model and geographical detectors, the factors influencing ANSP in small town areas were analyzed in terms of spatial spillover effects and the spatial stratified heterogeneity. The results showed a zigzagging downward trend of ANSP equivalent emissions over time. Spatially, the equivalent emissions of ANSP showed a distribution pattern of being high in the west and low in the east. There was a significant positive global spatial autocorrelation feature and there was an inverted “U-shaped” Environmental Kuznets Curve relationship between industrialization and ANSP. Affluence, population size, and cropping structure positively contributed to the reduction of ANSP. Population size, land size, and industrialization were highly influential factors affecting the spatial variation of ANSP and the interaction of these factors was bivariate or nonlinearly enhanced. This study provides a feasible reference for policymakers and managers to develop reasonable management measures to mitigate ANSP in small town areas during rapid industrialization.

## 1. Introduction

Agricultural non-point source pollution (ANSP) is prevalent globally, affecting 30% to 50% of the earth’s surface [1]; therefore, the study of ANSP has been a hot topic of academic research. Since the implementation of China’s reform and opening-up policy in 1978, industrialization has accelerated technological progress in agricultural production and large-scale mechanized agricultural production has been realized, leading to a substantial increase in agricultural production efficiency [2]. China’s agricultural economy has progressed remarkably, with the production of China’s major agricultural products increasing exponentially or tens of times, most of which are among the highest worldwide, with grain, oilseeds, and meat all ranking first worldwide [3]. However, the great achievements of agriculture have come at the cost of environmental losses. Since the agricultural production system is still a crude production model with high inputs and outputs [4], there are many misconducts in agricultural production. For example, the drenching with pesticides and fertilizers, the excretion of livestock and farming, and the uncontrolled discharge of rural household waste. A large amount of pollutant emissions is generated in this process, especially ANSP [5]. This not only gradually erodes the agroecological environment but also has a substantial impact on food security [6,7].

ANSP can damage the intrinsic structure of agricultural soils and accelerate their nutrient loss, resulting in increased soil consolidation; it can also lead to a decrease in the quality of agricultural products, such as excessive nitrate content in vegetables [8,9]. When the problem of ANSP is serious, it will lead to the phenomenon of the eutrophication of water bodies, destabilizing underwater ecosystems, and threatening the safety of potable water for humans and animals [10]. Therefore, increasing studies have been devoted to ANSP reduction strategies to achieve healthy and sustainable agricultural development.

Most studies that investigated ANSP have focused on units at the watershed [11], provincial [12], and municipal [13] levels, neglecting studies on small town areas. A small town is the extent of the built-up area in which an established town is located. It is the administrative, economic, and cultural center of the rural area, as well as the smallest administrative unit in China [14]. With the rapid development of industrialization and urbanization, big cities have been forced to balance the interests of economic development and the ecological environment, increasingly enforcing strict environmental protection laws and regulations, while small towns are relatively weak and lax in environmental protection. Thus, major polluters in big cities gradually move to small town areas, and some old technologies that were eliminated after technological upgrades in big cities are then transferred to small towns [15]. However, compared with large cities, small towns have a weak economic base and poor environmental management capacity, leading to serious ecological damage in small town areas [16], especially serious problems with ANSP.

Compared with point source pollution, ANSP is characterized by decentralization, randomness, extensiveness, difficulty in monitoring, and lagging, making it a challenge to measure [17,18]. Model simulation is a common method for monitoring and assessing ANSP and existing models can be divided into physical and empirical models. Physical models are constructed based on the intrinsic mechanisms of ANSP formation, simulating the process of pollutant generation and transport; then, ANSP loads are calculated through mathematical models. Physical models mainly include ANSWERS [19], SWRRB [20], SWAT [4], HSPF [21], and other models, which are mostly applicable to watershed-scale studies [22]. The empirical model is based on the data of each pollution unit and establishes the relationship between pollution units and ANSP emissions through multivariate linear correlation analysis; it then calculates the ANSP load of different units. Subsequently, the ANSP loads of each unit are summed to estimate the total ANSP emissions in the entire study area [23]. The empirical models mainly include the output coefficient model and non-point source inventory method. Compared with physical models, empirical models require less data and are more commonly applied [24,25]. Existing studies have shown that the application area of output coefficient models can be either a watershed or an administrative unit [11].

In the study of factors influencing ANSP, more scholars have focused on the impact of economic development on ANSP and the most frequently applied model is the Environmental Kuznets Curve (EKC) [26,27]. Most existing studies support the EKC hypothesis of ANSP [28,29] and the relationship between ANSP and economic growth has an inverted “U-shaped” distribution in most cases [30], where the pollution level increases with economic growth at low income levels and decreases with economic growth at high income levels. A small number of studies have also found that some regions conform to the “N” or inverted “N” distribution [29,31]; at the same time, some scholars have questioned the EKC [32]. In addition, some studies have used the logarithmic mean Divisia index factor decomposition method to deconstruct the effect of economic development on ANSP into different effects [33,34]. For example, Liang et al. [35] decomposed the effect of economic development on ANSP into scale, structural, and pollution reduction effects; Wu et al., [36] referring to the proposed IPAT model, decomposed the impact of economic growth on environmental pollution into three aspects: population size, affluence, and technical level. There are also studies that analyze its association with ANSP in terms of natural factors [37], financial development [38], and labor quality [12] as well as those that analyze their effects on ANSP from micro perspectives such as farmers’ behavior [39] and willingness [40].

The spatial spillover effect of environmental pollution refers to the first law of geography—the geographical proximity effect—which leads to the rapid expansion of pollutants with easy mobility to neighboring areas or even farther away, manifesting as a spatially contagious relationship [41,42]. ANSP is a “spillover” pollutant with a strong spatial spillover effect [42]. Existing studies on the influencing factors of ANSP are usually divided into different spatial units based on administrative boundaries, assuming that each regional unit is an independent individual, ignoring the spillover effects of ANSP between regions [43]. In contrast, the spatial econometric model assumes the existence of a spatial correlation among regions and considers the influence of surrounding areas on the study area, which can more fully reveal the effect of each influencing factor on ANSP [12].

Therefore, this study took Gongyi City, a typical representative of small towns under the background of rapid industrialization, as the research area. Based on the panel data set of 14 small towns in Gongyi City from 2002 to 2019, the non-point source inventory method that is more suitable for this study area was selected as the calculation method of ANSP load in Gongyi City. Furthermore, based on the spatial econometric model and the EKC hypothesis, the impact of various influencing factors on ANSP was empirically explored by selecting factors such as the degree of industrialization. At the same time, the driving factors behind the spatial stratification heterogeneity of ANSP, as well as the interaction between factors, were analyzed by using factor detection and interaction detection in geographical detectors. The research results can provide theoretical and practical guidance for the coordinated development of agricultural sustainable development planning in small town areas, thus enriching the existing research contents of regional development and economic geography.

The main contributions of this study are as follows: (1) The EKC model was extended to the spatial Durbin model to empirically explore the nonlinear relationship between industrialization and ANSP. Most existing studies directly assume that there is a linear relationship between industrialization and environmental pollution [44]. Given that industrialization is at a new stage of development from “high speed” to “high quality” [45], the impact of industrialization on ANSP may have a nonlinear relationship. (2) Most of the existing studies are based on traditional econometric models to analyze the factors affecting ANSP, ignoring the impact of possible spatial spillover effects and spatial heterogeneity of ANSP on the model estimation results [46,47]. In this study, the spatial spillover effect and spatial heterogeneity of ANSP were tested and estimated using spatial econometric models and geographical detectors.

## 2. Materials and Methods

### 2.1. Overview of the Study Area

Gongyi City is bound by latitude 34°31′~34°52′ N and longitude 112°49′~113°17′ E. It has a total area of 1041 km^2^ and is subordinate to Zhengzhou City of Henan Province. The topography is high in the southeast and low in the northwest, with the Song Mountains to the south and the Yellow River to the north. Gongyi City is in a temperate monsoon climate with high temperatures and rain in the summer and a cold and dry winter, which is suitable for growing crops.

Gongyi City is an important origin of Chinese township enterprises and a typical representative of a rapidly industrializing inland region [48]. From 1978 to 2019, the average annual growth rate of the secondary industry in Gongyi was 16.46% and the average annual share of the secondary industry was 50.37%, which was higher than the national average in the same period. The average annual growth rate in the secondary industry was also higher than that in Suzhou, a typical rapidly industrializing region in the east, in the same period. Therefore, Gongyi City can be considered a typical fast industrialization area [49]. In summary, in the context of rapid industrialization, this study of the evolution and influencing factors of ANSP in small towns, which takes Gongyi city as the study area, is of guiding significance for the sustainable development of agriculture in a large number of small town areas that are in or about to be in a rapidly industrializing area.

**Figure 1:**
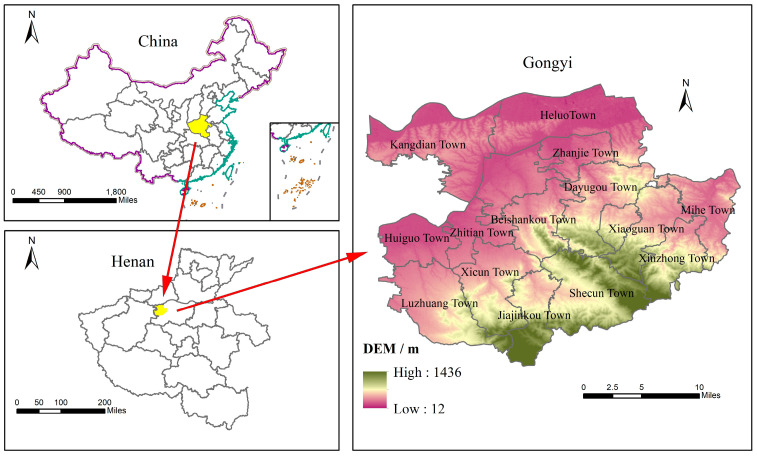
Map of the locations of 14 townships in Gongyi City.

Gongyi City has 5 streets and 15 townships. The streets are Xinhua Road Street, Dufu Road Street, Zijing Mountain Street, Yong’an Road Street, and Xiaoyi Street. The townships are Jizuankou Town, Luzhuang Town, Huiguo Town, Zhanjie Town, Zhitian Town, Zhulin Town, Shibucun Town, Mihe Town, Xinzhong Town, Hailuo Town, Kangdian Town, Dayugou Town, Xicun Town, Xiaoguan Town, and Beishankou Town. Owing to the absence of multi-year data in Zhulin Town during the study period, as well as the lack of statistics related to agricultural production and rural life in five of the streets, the remaining 14 small towns were selected as the study area in this research (Figure 1).

### 2.2. Research Methodology

#### 2.2.1. Accounting for ANSP

Considering both the reality of agricultural development in Gongyi City and the statistical yearbook data, this study categorized the four main sources of ANSP as agricultural fertilizer, agricultural solid waste, livestock and poultry breeding, and rural living (Table 1). All pollutants are calculated in terms of total nitrogen (TN), total phosphorous (TP), and chemical oxygen demand (COD) for measurement and comparison on a unified scale. The non-point source inventory method was used to measure ANSP in Gongyi City [26] and the relevant coefficients were calculated by referring to the existing literature [44,50,51]. The formula is as follows:

(1) $$E_{i}=\sum_{j}U_{ij}\times P_{ij}\times C_{ij}.$$

In Formula (1), *E_i_* is the pollutant emission of ANSP *i*, *U_ij_* is the indicator statistic of the *j*-th item in the inventory on pollutant *i*, *P_ij_* is the pollution production coefficient of the *j*-th item in the inventory on pollutant *i*, and *C_ij_* is the loss coefficient of the *j*-th item in the inventory on pollutant *i*. Determined by the pollution source and spatial characteristics, the loss coefficient indicates the combined effects of the regional ecological environment, precipitation, hydrology, and various management measures on ANSP.

Since the discharge standards and hazard levels of each pollutant vary, it was not possible to compare the discharge of various pollutants on the same scale; therefore, the concept of equivalent discharge was introduced to compare the discharge of different pollutants. The equivalent discharge of each pollutant was calculated uniformly according to the Class III standard in the Environmental Quality Standard for Surface Water (GB3838-2002) [51]. The formula is as follows:

(2) $$Q_{i}=E_{i}/S_{i}.$$

where *Q_i_* is the equivalent pollution load of pollutant *i*; *E_i_* is the pollutant discharge of pollutant *i*; and *S_i_* is the evaluation standard. COD was 20 mg/L, TN was 1 mg/L, and TP was 0.2 mg/L.

#### 2.2.2. Spatial Autocorrelation

The premise of the spatial econometric model used was the existence of significant spatial autocorrelation of the explanatory variables; therefore, this study first used the spatial autocorrelation model to analyze the equivalent emissions of ANSP in Gongyi City. The formula is as follows:

(3) $$I=\frac{n\sum_{i=1}^{n}\sum_{j\neq 1}^{n}W_{ij}\left(y_{i}-\bar{y}\right)\left(y_{j}-\bar{y}\right)}{\sum_{i=1}^{n}\sum_{j\neq 1}^{n}W_{ij}\sum_{i=1}^{n}{\left(y_{i}-\bar{y}\right)}^{2}}.$$

where *I* is Moran’s *I* for the equivalent emissions of ANSP; *n* denotes the number of township units; *y_i_* and *y_j_* are the equivalent emissions of ANSP in neighboring areas *i* and *j,* respectively; *y* is the average of the observed value *y_i_*; and *w_ij_* is the spatial weight matrix. The value range of *I* was −1 to 1. When *I* was close to 1, it indicated that the ANSP in Gongyi City was positively correlated in space; when *I* was close to −1, it indicated that the spatial correlation of ANSP was negative. When *I* approached 0, there was no spatial autocorrelation. The larger the absolute value of *I*, the stronger the spatial correlation of ANSP.

#### 2.2.3. Spatial Econometric Model

(1) Spatial measurement model construction and selection

To study the spatial effects of industrialization on ANSP, three spatial panel models—the spatial lagged model (SLM), the spatial error model (SEM), and the spatial Durbin model (SDM)—were constructed. These models are the most used because they focus on the spatiotemporal effects of the dependent variable and therefore have more accurate estimation results [52]. In this study, we selected the appropriate spatial panel model with corresponding fixed effects based on the estimation and testing framework for spatial panel econometric models proposed by Elhorst [53]. Among them, the expression of the SDM is:

(4) $$y_{it}=\beta \sum_{j=1}^{n}w_{ij}y_{jt}+x_{it}\gamma +\alpha \sum_{j=1}^{n}w_{ij}x_{jt}+u_{i}+v_{t}+\varepsilon_{it}.$$

where *y_it_* and *x_it_* denote the dependent and independent variables of the *i*-th township in year *t*, respectively; *β* is the spatial lag coefficient of the dependent variable; *γ* is the estimated coefficient of the independent variable; *α* is the spatial spillover coefficient of the independent variable; *W* denotes the spatial matrix; *u_i_* and *v_t_* denote the spatial and temporal effects, respectively; and *ε_it_* denotes the disturbance term obeying independent distribution. When *α* = 0 and *β* ≠ 0, Equation (4) was simplified to SLM and the model did not include the interaction effects of the explanatory variables. When *α* + *βγ* = 0, Equation (4) was simplified to SEM and the SEM considers that the spatial effects exist in the perturbation error terms; the implication of this is the error shock on these factors from the changes in the explanatory variables in the neighboring regions, which will have spatial effects on the regional explanatory variables.

(2) Weighting matrix

The model in this study was a spatial model that must portray the degree of inter-regional dependence. The degree of interdependence between regions is usually characterized by a spatial weight matrix. To increase the robustness, this study analyzed the 0–1 adjacency spatial weight matrix as the basis and the robustness of the geographic distance spatial weight matrix was checked, which uses the inverse function of the distance between two places as the elements in the matrix. The formula is as follows:

(5) $$W_{1}=\left\{\begin{array}{l} 1,\;i=j\\ 0,\;i\neq j \end{array}\right..$$

Whether the spatial locations are adjacent is the basis for judging the 0–1 adjacency space weight matrix. If two regions are adjacent, *W*_1_ = 1; if they are not adjacent, *W*_1_ = 0:

(6) $$W_{2}=\left\{\begin{array}{c} 1/d,\;i\neq j\\ 0,\;i=j \end{array}\right..$$

where *d* is the distance between the locations of the two regional geographic centers.

(3) Selection of influencing factors

Referring to relevant studies on the analysis of factors influencing ANSP, it can be found that scholars’ selection of factors influencing ANSP mostly revolves around industrialization, affluence, population size, planting scale, and planting structure [52,54,55,56,57]. Combined with the development background of rapid industrialization in Gongyi city, this study used industrialization as the core explanatory variable. With the rapid development of industrialization, relatively high wage levels attract young and strong rural laborers from the agricultural sector to the industrial sector, and according to the theory of induced technological change, when labor is insufficient in the agricultural production process, farmers tend to choose chemical technologies that can replace labor and are relatively inexpensive, exacerbating agricultural surface source pollution [58]. Furthermore, the non-agriculturalization of arable land is an inevitable phenomenon in the process of industrialization and construction. In this process, the area of arable land decreases, the quality decreases, and the degree of intensification increases, which in turn has a certain impact on ANSP [59]. At the same time, the development of industrialization is conducive to promoting technological progress in agricultural production, and the development and promotion of some environmentally friendly technologies have reduced the generation of ANSP to a certain extent. Thus, it can be seen that the impact of industrialization on ANSP is uncertain. Therefore, based on the spatial econometric model, this study introduced the squared term of industrialization to empirically analyze the EKC of industrialization on ANSP. Considering that all towns in Gongyi City are dominated by industry and agriculture, this study expressed the degree of industrialization by dividing the total industrial output value by the total industrial and agricultural output value.

Affluence level, population size, planting size, and planting structure were selected as control variables (Table 2). The level of per capita net income of farmers was used to characterize the affluence of small town areas. The level of affluence mainly refers to the level of economic development of an area. The level of economic development determines people’s productivity and lifestyle, such as the method of land use, agricultural production and operation, and residents’ awareness of environmental protection. When the level of economic development is low, driven by economic interests, people will pursue the growth of output and income and adopt rough agricultural production and management methods, resulting in an increase in ANSP emissions. At higher levels of economic development, people’s higher awareness of environmental protection induces agricultural producers to improve production methods and reduce ANSP emissions. Therefore, the economic development levels at different stages will have different effects on ANSP emissions.

The population size factor was expressed in terms of the population density. The impact of the population size on ANSP is mainly analyzed from two perspectives: agricultural production and operation and rural life. The rural population is the main participant in agricultural economic activities, and the larger the size of the rural population in agricultural production and operation, the greater the demand for agricultural raw materials and the greater the input of agricultural chemicals, resulting in more serious ANSP. However, some scholars argue that population growth leads to an increased demand for resources, forcing people to improve production techniques, find substitutes, and increase efficiency, making the impact of population growth on the environment neutral or even positive [60]. Rural domestic pollution emissions are one of the components of ANSP, involving the discharge of domestic sewage, domestic waste, and human waste. The larger the population living in the area, the greater the likelihood of causing ANSP. Therefore, there is uncertainty in whether the impact of population size on ANSP is positive or negative.

The sown area per capita is used to characterize the scale of cultivation. As a proxy for the supply status of demand for agricultural resources in economic development, this indicator can reflect the scarcity of agricultural resources. On the one hand, the larger the sown area per capita, the richer the agricultural resources in the region, and less pressure is placed on intensive land operations. This creates prerequisites for land transfer and is conducive to promoting large-scale land operations. According to the economies of scale theory, a moderate-scale operation can improve the utilization rate of agricultural production factors and effectively reduce ANSP emissions [61]. On the other hand, a larger sown area per capita also indicates that more agricultural production factor inputs are required, resulting in an increase in ANSP emissions. Therefore, there is uncertainty in whether the planting scale has a positive or negative impact on ANSP.

The ratio of the sown area of cash crops to the sown area of food crops was used to characterize the crop planting structure. The crops planted by farmers mainly include two types of crops: food crops and cash crops. There are large differences in the types and quantities of agricultural production materials, such as the fertilizer demanded by different types of crops; for example, vegetables and wheat in food crops require large amounts of water and fertilizer in agricultural cultivation, while fruit trees and cotton in cash crops have relatively little demand for chemical fertilizer [30]. Therefore, the crop cultivation structure is an important factor affecting ANSP and changes in the structure will lead to large changes in ANSP emissions.

#### 2.2.4. Geographical Detectors

Although spatial econometric models consider spatial factors in ANSP, they lack an explanation for the spatial stratified heterogeneity in ANSP. Spatial stratified heterogeneity refers to the fact that an attribute varies between types or regions [62]. Geographical detectors can detect the spatial stratified heterogeneity of the explanatory variables to some extent [63], overcoming the limitations of the mechanisms of interaction among variables in traditional analysis methods and more clearly testing the magnitude of the effect of univariate or bivariate factors on the influence of the dependent variable [64]. Therefore, in this study, the factor and interaction detectors in the geographical detectors were adopted to quantitatively analyze the driving factors of ANSP spatial differentiation in Gongyi City and the interactions among the factors. The calculation equation is as follows:

(7) $$q=1-\frac{\sum_{h=1}^{L}N_{h}\sigma_{h}^{2}}{N\sigma^{2}}.$$

where *q* denotes the explanation of the driving factor, which ranged from [0, 1], where the closer the variable was to 1, the greater its explanatory power; *h* is the stratification of the explanatory variables *Y* or *X*; *N_h_* and *N* are the number of cells in stratum *h* and the whole region; *σ_h_^2^* and *σ^2^* represent the variance of the values in stratum *h* and the whole region *Y*, respectively.

The interaction detector determines the characteristics of the interaction between bivariate factors by comparing the *q* value of a single factor and the *q* value of a two-factor interaction. The method is advantageous because the assumptions of the interaction are not limited to those of traditional statistical methods. The interaction of the driver is identified by the *q*(*x_i_*∩*x_j_*) value of the detection result to determine whether the joint action between the drivers increases or weakens the explanatory power of the analyzed variables. The interaction detection results can be classified into five categories: nonlinearly enhanced, independent, two-factor enhanced, single-factor nonlinearly diminished, and nonlinearly diminished.

### 2.3. Data Sources and Processing

The statistical data in this research were mainly from the *Statistical Yearbook of Gongyi City* and the *Statistical Bulletin of National Economic and Social Development of Gongyi City* during 2002–2019. Linear interpolation was used to complete individual missing values in the selected panel data. In order to eliminate the influence of price factors, the relevant data were deflated, with 2002 as the base period. The correlation coefficients used in the accounting of ANSP were mainly from documents such as the *Handbook on Fertilizer Loss Coefficients of Agricultural Pollution Sources*, *Emission Standards for Pollutants in Livestock and Poultry Breeding* (GB18596-2001), *Handbook on Agricultural Technology and Economy*, the *Handbook on Production and Discharge Coefficients of Urban Domestic Pollution Sources*, and the research results of relevant studies. In addition, in the spatial econometric model, logarithmic treatment was applied to ANSP equivalent emissions, farmers’ net income per capita, population density, sown area per capita, and the crop cultivation structure to reduce the absolute values of variables and eliminate the heteroskedasticity among variables as much as possible. Stata 16.0 software was used to establish a mixed regression model and calculate the variance inflation factor (VIF). The mean value of VIF among the five explanatory variables was 1.44 and the maximum value was 2.02; all variables were less than 10, indicating that there was no significant multicollinearity.

## 3. Results

### 3.1. Analysis of the Spatiotemporal Evolution of ANSP

#### 3.1.1. Time-Series Evolution of ANSP

**Figure 2:**
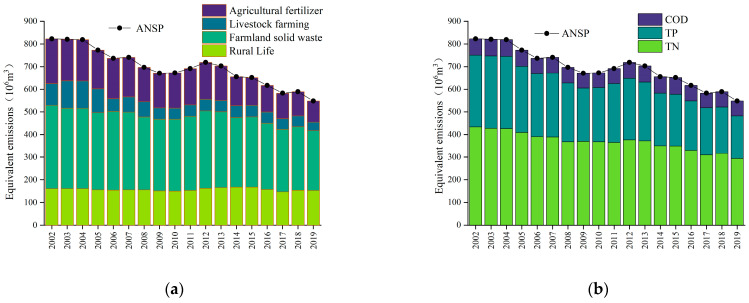
(**a**) ANSP and its pollution sources; (**b**) ANSP and its pollutants.

The equivalent emissions of ANSP and its sources and pollutants in Gongyi City were calculated based on the non-point source inventory method and the results are shown in Figure 2. Overall, the equivalent emissions of ANSP in Gongyi City during 2002–2019 show a zigzagging decreasing trend from 8.23 × 10^8^ m^3^ in 2002 to 5.48 × 10^8^ m^3^ in 2019, indicating that the management of ANSP in Gongyi City displayed a positive trend. From the source equivalent emissions, all four sources of pollution equivalent emissions showed a decreasing trend: from 2002 to 2007, agricultural solid waste > agricultural fertilizer > rural life > livestock and poultry farming; and from 2008 to 2019, agricultural solid waste > rural life > agricultural fertilizer > livestock and poultry farming. As can be seen from the contribution rate of pollution source equivalent emissions, the proportion of rural living emissions increased from 19.58% in 2002 to 30.93% in 2019; the proportion of pollution emissions from farmland solid waste increased from 44.72% in 2002 to 48.16% in 2019; the proportion of pollution emissions from agricultural fertilizer decreased from 24.09% in 2002 to 17.05%; and the proportion of livestock and poultry breeding emissions decreased from 11.62% in 2002 to 6.89% in 2019. In terms of pollutant equivalent emissions, the equivalent emissions of TN, TP, and COD all gradually decreased, and the overall order was TN > TP > COD. From the contribution of pollutant equivalent emissions, it can be seen that the proportion of TN emissions increased from 52.73% in 2002 to 53.62% in 2019; the proportion of TP emissions decreased from 38.55% in 2002 to 34.33% in 2019; and the share of COD emissions increased from 8.72% in 2002 to 12.05% in 2019.

#### 3.1.2. Spatial Evolution of ANSP

**Figure 3:**
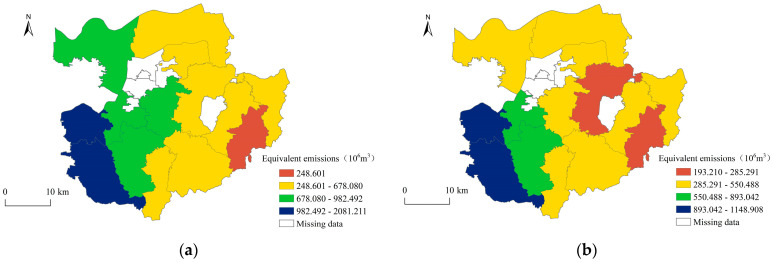
Spatial distribution of ANSP in Gongyi City: (**a**) 2002; (**b**) 2019.

The natural breakpoint method was applied in ArcGIS 10.2 to classify the ANSP equivalent emissions of each township into four categories in 2002 and 2019 (Figure 3). It can be seen that ANSP in Gongyi City has significant spatial heterogeneity, with a spatial distribution pattern that is high in the west and low in the east. The areas with higher ANSP equivalent emissions are concentrated in Huiguo and Luzhuang Towns in the west, which have larger rural populations and a better agricultural resources endowment, a larger agricultural production scale, and higher ANSP equivalent emissions. The areas with lower emissions of ANSP are concentrated in the eastern areas of Xinzhong Town and Dayugou Town, which are typical mountainous towns with sparse rural populations and relatively regressive agricultural development.

#### 3.1.3. Spatial Correlation Analysis of ANSP

Stata 16.0 software was used to test the spatial correlation of ANSP equivalent emissions in 14 small town areas in Gongyi City from 2002 to 2019 (Table 3). The results show that Moran’s I index of ANSP equivalent emissions is significantly positive during the study period and the change trend also indicates Moran’s I index is increasing, demonstrating that ANSP in each township of Gongyi City is not randomly spatially distributed but has positive global spatial autocorrelation characteristics. Over time, the agglomeration gradually increased.

### 3.2. Spatial Econometric Model Empirical Analysis

Given that spatial econometric models have different forms, this study used Stata16.0 to perform LM, Wald, LR, and Hausman [30,65] tests. The results are shown in Table 4.

As can be seen from Table 4, the LM test and R–LM test results reject the original hypothesis at the 5% significance level, indicating that the model has both spatial lag and spatial error terms. The LR test and Wald test results reject the original hypothesis at the 1% significance level, indicating that the SDM cannot be reduced to a spatial lag model and SEM, i.e., the SDM is optimal for the simulation of ANSP in Gongyi City. The SDM is optimal for the simulation of influencing factors. Meanwhile, the Hausman test results show that the original hypothesis of random effect is rejected at the 1% significance level. Therefore, the fixed effects model of the SDM was selected for the analysis and the regression results are shown in Table 5. The fixed effects model contains three types of effects: time fixed, spatially fixed, and spatiotemporally fixed. In order to compare which fixed effects model has the best fitting effect, based on the magnitude of the log-likelihood and dispersion (sigma^2^) in Table 6, the spatiotemporally fixed effects’ fitting results were optimal. Therefore, the spatiotemporally fixed SDM model was finally selected as the analytical model in this study.

#### 3.2.1. Decomposition of Spatial Spillover Effects

The estimated coefficients of the model could not explain the spatial spillover effects of ANSP. Therefore, a partial differential solution was calculated based on Lesage’s study to assess the magnitude of the influence and spillover effects of the explanatory variables through direct and indirect effects [66] and the results are shown in Table 6. The direct effect is expressed as the magnitude of the effect of the explanatory variables in the region on the explanatory variables in the region; the indirect effect is expressed as the effect of the explanatory variables in neighboring regions on the explanatory variables in the region; and the total effect is expressed as the sum of the direct and indirect effects.

There is an inverted “U-shaped” EKC relationship between industrialization and ANSP. As can be seen from Table 7, the total effect of the primary term of industrialization is positive, the total effect of the secondary term is negative, and both pass the 1% significance test. Thus, as industrialization progressed, ANSP first increased and then decreased. With rapid industrialization, the relatively high wage level attracts young and strong rural laborers to transfer from the agricultural sector to the industrial sector, which leads to the emergence of part-time farming behavior. According to the theory of induced technological change, when there is a shortage of labor in the agricultural production process, farmers tend to choose chemical technologies that can replace labor and are relatively cheap, exacerbating ANSP [67]. Furthermore, the non-agriculturalization of arable land is an inevitable phenomenon in the process of industrialization construction, in which the area of arable land decreases, the quality decreases, and the degree of intensification increases, which in turn has a certain impact on ANSP [68]. At the same time, the technological progress brought on by industrialization has accelerated the evolution of traditional agriculture to modern agriculture (i.e., petrochemical agriculture), and agricultural development has entered a “chemical trap” with an increasing number of chemical products being applied to agricultural production [69]. All these effects have exacerbated ANSP. When industrialization crosses the inflection point, the positive externalities of industrialization play a dominant role and the growth of national fiscal revenue provides sufficient financial support for ANSP control. At the same time, the development of efficient and low-consumption environmental protection technologies and advanced management technologies also makes the prevention and control of ANSP more complete. The indirect effect was not significant, indicating that the increase in the industrialization level in neighboring townships had no significant effect on ANSP in this township.

From the decomposition of the effects of each control variable, affluence played a positive role in promoting ANSP. The direct effect of the net per capita income of farmers on ANSP was positive and passed the test at the 5% significance level, indicating that increasing the rural economy level played a driving role in ANSP in this township. This may be because as affluence increases, increasing factors of production, such as chemical fertilizers, pesticides, and agricultural films are put into agricultural production, which in turn increases ANSP emissions. The indirect effect was positive; however, it did not pass the significance test. This indicates that the increase in the economic level of neighboring townships had a positive effect on the ANSP in the township.

Population size plays a positive role in promoting ANSP. The direct effect of the rural population density on ANSP was positive and passed the test at the 1% significance level, indicating that the increasing rural population density played a facilitating role in ANSP in this township. This is because the rural population is the main participant in agricultural economic activities and rural life; therefore, a higher population density leads to increased pressure on the agricultural environment in this township; a higher population density means that human production, living, and consumption activities in the area will all increase, and the accompanying emissions of ANSP will also increase [12]. The indirect effect of the rural population density on ANSP was negative and insignificant, indicating that, although there is some difference in the rural population density within each township, this difference did not trigger population movement in neighboring townships, which in turn has an impact on ANSP emissions in this township.

The effect of the planting scale on ANSP was not significant. The direct effect of the per capita sowing area on ANSP was positive and passed the test at the 1% significance level, indicating that increasing the per capita sowing area played a driving role in ANSP in this township. This is likely because most townships in Gongyi City are located in mountainous and hilly areas, with poor agricultural production resources and generally small land operation scales; to meet the social demand for agricultural products, they will increase the land intensification degree, increasing the input of pesticides, fertilizers, and other production materials, which to a large extent aggravates ANSP. The indirect effect was negative and passed the test at the 10% significance level, indicating that the increase in the per capita sown area in neighboring townships had a suppressive effect on the ANSP in this township. This is because the higher planting scale of neighboring townships will produce a clustering effect, attracting the inflow of agricultural production resources from this township to the neighboring area, which in turn reduces the ANSP emissions in this township. The total effect was not significant, likely because the direct and indirect effects cancelled each other out.

There is a significant positive contribution of the planting structure to the ANSP. The direct effect of the cropping structure is positive and passes the test at the 1% significance level, indicating that the increase in the proportion of the sown area of cash crops will promote the growth of ANSP emissions in this township. As the level of economic development increases, farmers pursue higher profits in agricultural production and operation, resulting in a decrease in the proportion of the sown area of grain in the plantation industry and an increase in the sown area of cash crops. While cash crops bring farmers rich economic returns, they also produce more serious ANSP. In terms of the amount of fertilizer applied, it can be seen from the 2010 Guidance on Scientific Fertilization of Major Crops that the amount of fertilizer applied to cash crops is significantly higher than that of food crops. The average utilization rate of nitrogen fertilizer on farmland planted with vegetables, fruit trees, and flowers only reached approximately 10%, which is much lower than the utilization rate of 35% for food crops [36]. The indirect effect was positive but failed the significance test, indicating that the planting structure of neighboring townships was exemplary; however, the spillover effect of this demonstration was not significant and had a very limited impact on ANSP emissions in this township.

#### 3.2.2. Robustness Test

A reasonable measure of the spatial relationship between regions is a prerequisite for spatial econometric analysis and the construction of a spatial weight matrix is a key step. A subjective and single spatial weight matrix may not truly reflect the complex relationships among different regions [70]. Therefore, the accuracy and robustness of the spatial econometric modeling results are highly dependent on the spatial weight matrix. To ensure the reliability of the above empirical results, the model was tested by replacing the weight matrix. The direct, indirect, and total effects under the SDM model of spatiotemporally fixed effects based on the geographic distance weight matrix are shown in Table 7. Compared with the 0–1 adjacency weight matrix, the regression coefficients and significance changes of each effect based on the geographic distance weight matrix were smaller, indicating that the results of this study were robust.

The model may have endogeneity and omitted variables that lead to biased estimation results since ANSP is a dynamic and continuous process; consequently, the current increase in ANSP will be influenced by both current- and prior-period factors. Therefore, a dynamic spatial Durbin model (DSDM) with a first-order lag containing the explanatory variable ANSP was simultaneously constructed. The model results are shown in Table 8, and the results indicate that the direction and significance of the regression coefficients are basically consistent with those of previous studies after considering endogeneity. Furthermore, from the total effect of short-term and long-term effects, each influencing factor exhibits a stronger temporal effect of short-term effects than long-term effects.

### 3.3. Analysis of ANSP Drivers Based on Geodetectors

**Figure 4:**
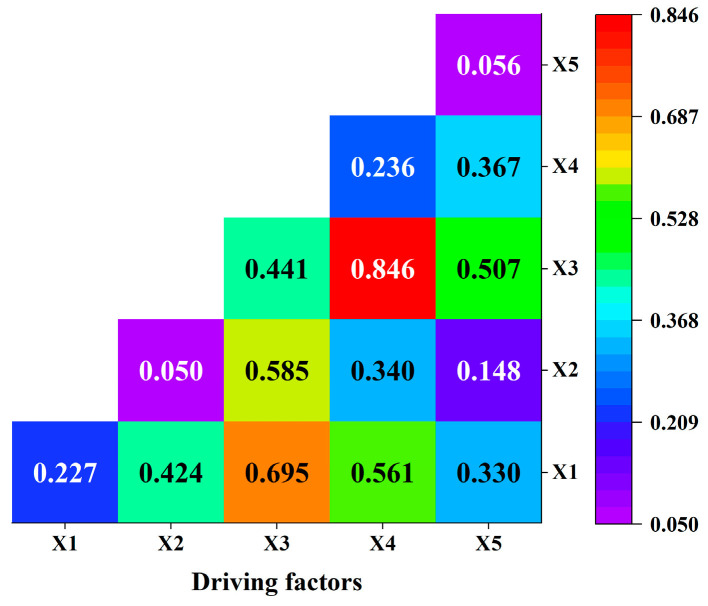
Interaction of ANSP detection factors.

To further explore the driving factors of spatially stratified heterogeneity of ANSP in Gongyi City, this study employed geodetector software for driving factor detection. In this study, the five detection factors of industrialization (X1), per capita net income of farmers (X2), population density (X3), per capita sown area (X4), and grain crop planting structure (X5) were selected from five dimensions of industrialization, affluence, population size, land size, and planting structure to analyze the spatial heterogeneity of ANSP in Gongyi City. RStudio software and the R language “GD” package were used to detect the geographic probe factors (Table 9) and interactions (Figure 4).

Table 9 shows that the spatial heterogeneity of ANSP is mainly the result of the combined effect of multiple factors and the driving factors are in the order: X3 > X4 > X1 > X5 > X2. This indicates that the population size, land size, and industrialization degree were the most influential factors affecting ANSP in Gongyi City. In the process of ANSP management in small town areas in the future, it is more important to strengthen the population and land planning to reduce the pressure of the environmental carrying capacity. The design and promotion of agricultural green production technology should be accelerated in the meantime, crossing the industrialization EKC inflection point as early as possible, releasing the positive externalities of industrialization, reducing ANSP, and achieving green and sustainable agricultural development.

ANSP is the result of the joint action of multiple factors; therefore, the interaction factor exploration module was used for in-depth analysis (Figure 4). The interaction of the factors was bifactor-enhanced or nonlinearly enhanced, i.e., the influence of factors on the interaction was greater than the sum of the independent forces of two factors, reflecting the complex characteristics between the compound influencing factors and ANSP. The explanatory power of the interaction between the population density and the sown area per capita was significantly stronger than that of the interaction among other factors, which was the controlling factor for the significant spatial variation of ANSP.

## 4. Discussion and Recommendations

This study examined the evolution of ANSP and the factors influencing it in small town areas in the context of rapid industrialization from a geographic perspective. Although some scholars have already conducted related studies, this study still had some innovative features. First, in comparison to Mahmood et al. [59] who suggested that industrialization would lead to increased environmental pollution, this study showed an inverted “U” shaped EKC relationship between industrialization and ANSP in small towns in a typical area of ANSP in China. This study is helpful to clarify the relationship between industrialization and ANSP in small towns with contradictory agricultural development and to extend the boundary of environmental Kuznets theory to a certain extent. Furthermore, compared with the traditional regression model used by Zhang et al. [26] to analyze the influencing factors of ANSP, this study analyzed the influencing factors of ANSP in terms of both spatial spillover effects and spatial stratified heterogeneity, which was conducive to improving the credibility of the results. Meanwhile, it was found that affluence, population size, and cropping structure positively contributed to ANSP; these findings are consistent with those of existing studies [35,36,38]. The spatial spillover effect of industrialization on ANSP was not significant, which is likely due to the small scale of the study area in this study and the low exchange of resource factors between regions. The results of this study can provide a scientific basis for the prevention and the control of ANSP in similar rapidly industrializing small town areas and play a supporting role in sustainable agricultural development.

Based on the results of the empirical analysis, combined with the basic ideas of agroecological economics and sustainable agricultural development, the following suggestions for the prevention and control policies of ANSP are proposed.

(1) Control at the source to prevent the risk of ANSP is necessary. Temporally, ANSP in Gongyi City from 2002 to 2019 showed a zigzagging downward trend, indicating that the agricultural development in Gongyi City was positive; however, there are still some inefficiencies and environmental losses and there is substantial room for agricultural pollution reduction. From the perspective of pollution sources, ANSP emissions mainly come from farmland solid waste and rural life, livestock and poultry breeding, and agricultural fertilizer; pollutant emissions were mainly in the order of TN > TP > COD. Thus, real-time prediction and assessment of production and discharge sources of pollution is crucial. This will also improve the effective use of agricultural solid waste storage and processing equipment, promote the updating and upgrading of relevant technologies, and enhance the standardization of rural living environments via targeted pollution risk mitigation.

(2) Strengthening cooperation for mutual benefits and creating “eco-efficient” situations is key. According to the spatial econometric model results, ANSP shows a significant spatial correlation and clustering distribution pattern. Therefore, policymakers should fully consider the spillover effect of ANSP when formulating policies, eliminate local protectionism, conduct comprehensive planning from the perspective of regional integration, establish a complete agricultural policy linkage mechanism, and strengthen inter-regional agricultural production resource sharing, exchange, and cooperation.

(3) Increasing the research, development, and promotion of green agricultural production technologies will continue to be important. From analyzing the impact of industrialization on ANSP, we can see that the improvement of agricultural green production technology can effectively promote an early crossing of the inflection point of the EKC. Technological innovation is the only path for China’s ongoing agricultural development. In terms of China’s agricultural growth, although the contribution of science and technology to agricultural development has reached 42%, it is still far behind that of other developed countries (which tend to be closer to 70%). Therefore, we should increase investment in agricultural science and technology innovation and vigorously develop and promote new resource-saving and environmentally friendly agricultural technologies. For example, by promoting soil testing and fertilization techniques, encouraging farmers to apply more organic fertilizers, developing new pesticides such as bionic pesticides, and promoting biodegradable agricultural films. Furthermore, as China is limited by farmers’ education levels, learning abilities, and production input levels, there is a greater need to develop simple, easy-to-operate, and low-cost agricultural green production technologies.

In general, this study has some limitations. First, the non-point source inventory method was chosen for the measurement of ANSP, which means the research results may deviate from the actual situation. Second, the spatial Durbin model was used for the influence factor analysis, which lacked consideration of local spatial regression models. Therefore, in future studies on ANSP, field ANSP measurements should be increased as much as possible and local spatial regression models should be tried in the research method, so as to improve the accuracy of the research conclusions.

## 5. Conclusions

In this study, panel data from 14 small towns in Gongyi City from 2002–2019 were used to study the evolution of ANSP and influencing factors in small town areas in the context of rapid industrialization using the non-point source inventory method, spatial econometric model, and geographical detectors. The main conclusions are as follows.

(1) Temporally, ANSP in Gongyi City showed a zigzagging decreasing trend from 2002 to 2019, from 8.23 × 10^8^ m^3^ in 2002 to 5.48 × 10^8^ m^3^ in 2019. Pollution sources mainly came from solid farm waste and rural life, followed by livestock and poultry breeding and agricultural fertilizer; pollutant emissions were mainly in the order of TN > TP > COD.
(2) Spatially, the equal standard emissions of ANSP showed a distribution pattern of being high in the west and low in the east, with higher areas concentrated in the western Huiguo and Luzhuang Towns and lower areas concentrated in the eastern Xinzhong and Dayugou Towns. Meanwhile, there was a positive global spatial autocorrelation feature.
(3) There was an inverted “U-shaped” EKC curve between industrialization and ANSP in Gongyi City, i.e., as industrialization progressed, the equivalent emissions of ANSP first increased and then decreased; affluence, population size, and planting structure contribute positively to the reduction in ANSP. Furthermore, all influencing factors showed stronger short-term temporal effects.
(4) Population size, land size, and industrialization were the high-impact factors on ANSP in Gongyi City and the interactions between these factors showed a two-way or nonlinear enhancement.

## Figures and Tables

**Table 1:** List of ANSP in Gongyi City.

Source of Pollution	Pollution Unit	Metrics	Unit
Agricultural fertilizer	Nitrogen fertilizer	Application amount (discounted)	10,000 tons
	Phosphate fertilizer	Application amount (discounted)	10,000 tons
	Compound fertilizer	Application amount (discounted)	10,000 tons
Livestock farming	Pig	Year-end stockpile	10,000 heads
	Cow	Year-end stockpile	10,000 heads
	Sheep	Year-end stockpile	10,000 heads
	Poultry	Year-end stockpile	10,000 heads
Farmland solid waste	Wheat	Total production	10,000 tons
	Corn	Total production	10,000 tons
	Beans	Total production	10,000 tons
	Potatoes	Total production	10,000 tons
	Oil crops	Total production	10,000 tons
	Cotton	Total production	10,000 tons
Rural household activities	People	Rural population	10,000

**Table 2:** Explanatory variables description.

Variables	Indicators	Descriptions	Abbreviations
Industrialization	Degree of industrialization (million yuan)	Total industrial output value/Total industrial and agricultural output value	DI
Affluence	Per capita net income of farmers (yuan)	Per capita net income of farmers	PC
Population size	Population density (persons/km^2^)	Total population/land area	PD
Planting scale	Sown area per capita (ha/person)	Area sown to crops/total population	SAC
Planting structure	Crop cultivation structure	Area sown to cash crops/area sown to food crops	PS

**Table 3:** Moran’s I value of ANSP in Gongyi City.

Years	Moran’s I	Years	Moran’s I	Years	Moran’s I
2002	0.297 ***	2008	0.423 ***	2014	0.431 ***
2003	0.265 **	2009	0.382 ***	2015	0.425 ***
2004	0.265 **	2010	0.357 ***	2016	0.430 ***
2005	0.370 ***	2011	0.347 ***	2017	0.429 ***
2006	0.353 ***	2012	0.341 ***	2018	0.434 ***
2007	0.386 ***	2013	0.356 ***	2019	0.494 ***

Note: *** and ** denote significance at the 1% and 5% levels, respectively.

**Table 4:** Spatial econometric model correlation test results.

Inspection Method	Statistical Values	*p* Value	Inspection Method	Statistical Values	*p* Value
LM lag	22.100 ***	0.000	LR spatial lag	17.650 ***	0.007
LM error	6.088 **	0.014	Wald spatial error	23.730 ***	0.000
R-LM lag	91.542 ***	0.000	LR spatial error	22.650 ***	0.001
R-LM error	75.530 ***	0.000	Hausman	22.390 **	0.050
Wald spatial lag	18.080 ***	0.006			

Note: *** and ** denote significance at the 1% and 5% levels, respectively.

**Table 5:** Regression results of the SDM with different fixed effects.

Variables	Spatial Fixed Effects	Time Fixed Effects	Spatiotemporally Fixed Effects
DI	207.397 ** (2.09)	305.107 *** (3.87)	283.282 *** (3.75)
DI^2^	−110.187 ** (−2.15)	−161.088 *** (−3.91)	−150.049 *** (−3.81)
lnPC	0.261 *** (3.07)	0.055 (0.80)	0.195 ** (2.50)
lnPD	0.501 *** (12.13)	0.704 *** (7.14)	0.651 *** (6.93)
lnSAC	0.317 *** (4.75)	0.430 *** (6.12)	0.354 *** (5.10)
lnPS	−0.105 (−0.49)	0.504 *** (3.64)	0.652 *** (4.65)
W*DI	742.357 *** (3.60)	−87.740 (−0.66)	59.788 (0.43)
W*DI^2^	−393.937 *** (−3.69)	49.893 (0.72)	−28.925 (−0.33)
W*lnPC	1.141 *** (5.34)	−0.173 ** (−2.38)	0.272 (1.61)
W*lnPD	0.813 *** (7.53)	−0.238 (−1.24)	−0.148 (−0.64)
W*lnSAC	0.329 ** (2.12)	0.027 (0.23)	−0.173 (−1.28)
W*lnPS	1.032 ** (2.11)	0.222 (0.86)	0.551 * (1.92)
Spatialrho	−0.589 *** (−6.42)	−0.013 (−0.15)	−0.224 ** (−2.31)
sigma^2^	0.047 *** (10.70)	0.014 *** (11.22)	0.012 *** (11.14)
N	252	252	252
R^2^	0.163	0.297	0.108
Log-likelihood	16.066	179.209	196.371

Note: ***, **, and * indicate significance at the 1%, 5%, and 10% levels, respectively; numbers in parentheses are Asymptot t-stat values for the coefficients of the corresponding variables.

**Table 6:** SDM model spatial spillover effect decomposition.

Explanatory Variables	Direct Effect	Indirect Spillover	Total Effect
DI	284.495 *** (3.82)	−4.751 (−0.04)	279.745 ** (2.25)
DI^2^	−150.832 *** (−3.87)	4.965 (0.08)	−145.867 ** (−2.25)
lnPC	0.185 ** (2.32)	0.203 (1.32)	0.388 ** (2.22)
lnPD	0.666 *** (7.13)	−0.254 (−1.33)	0.402 ** (2.08)
lnSAC	0.368 *** (5.36)	−0.217 * (−1.84)	0.151 (1.24)
lnPS	0.642 *** (4.81)	0.375 (1.54)	1.017 *** (3.74)

Note: ***, **, and * indicate significance at the 1%, 5%, and 10% levels, respectively; numbers in parentheses are Asymptot t-stat values for the coefficients of the corresponding variables.

**Table 7:** Decomposition of the SDM model spatial spillover effects based on a geographic distance weight matrix.

Explanatory Variables	Direct Effect	Indirect Spillover	Total Effect
DI	266.600 *** (3.41)	−22.093 (−0.12)	244.507 (1.42)
DI^2^	−140.841 *** (−3.44)	16.940 (0.17)	−123.900 (−1.39)
lnPC	0.166 ** (2.09)	−0.106 (−0.56)	0.060 (0.33)
lnPD	0.751 *** (7.71)	0.174 (0.71)	0.925 *** (3.86)
lnSAC	0.420 *** (5.81)	−0.108 (−0.65)	0.313 ** (1.99)
lnPS	0.617 *** (4.57)	0.312 (0.83)	0.930 ** (2.49)

Note: ***, and ** indicate significance at the 1% and 5% levels, respectively; numbers in parentheses are Asymptot t-stat values for the coefficients of the corresponding variables.

**Table 8:** Spatial effect decomposition of the DSDM model.

Explanatory Variables	Short-Term Direct Effects	Short-Term Indirect Spillover	Short-Term Aggregate Effect	Long-Term Direct Effects	Long-Term Indirect Spillover	Total Long-Term Effect
DI	257.552 *** (3.11)	38.991 (0.28)	296.543 ** (1.36)	263.464 *** (2.99)	−24.922 (−0.20)	238.543 ** (1.98)
DI^2^	−136.704 *** (−3.16)	−17.698 (−0.24)	−154.402 ** (−1.97)	−140.086 *** (−3.03)	15.884 (0.24)	−124.202 * (−1.98)
lnPC	0.151 * (1.85)	0.177 (1.16)	0.328 * (1.87)	0.142 * (1.67)	0.122 (0.91)	0.263 * (1.89)
lnPD	0.722 *** (7.69)	−0.013 (−0.06)	0.709 *** (3.26)	0.748 *** (7.28)	−0.178 (−0.90)	0.570 *** (3.30)
lnSAC	0.377 *** (5.30)	−0.144 (−1.22)	0.233 ** (1.79)	0.402 *** (5.29)	−0.215 ** (−1.99)	0.187 * (1.80)
lnPS	0.674 *** (4.92)	0.463 * (1.78)	1.137 ** (3.87)	0.660 *** (4.61)	0.254 (1.11)	0.914 ** (3.93)

Note: ***, **, and * indicate significance at the 1%, 5%, and 10% levels, respectively; numbers in parentheses are Asymptot t-stat values for the coefficients of the corresponding variables.

**Table 9:** The intensity of action of ANSP detection factor *q*.

Variables	X_1_	X_2_	X_3_	X_4_	X_5_
*q*-value	0.227	0.050	0.441	0.236	0.056
*p*-value	0.000	0.048	0.000	0.000	0.013

## Data Availability

The associated dataset of the study is available upon request to the corresponding author.
